# Value of high frame rate contrast enhanced ultrasound in gallbladder wall thickening in non-acute setting

**DOI:** 10.1186/s40644-023-00651-x

**Published:** 2024-01-08

**Authors:** Lianhua Zhu, Nan Li, Yaqiong Zhu, Peng Han, Bo Jiang, Miao Li, Yukun Luo, Dirk-André Clevert, Xiang Fei

**Affiliations:** 1 Department of Ultrasound, the First Medical Center, Chinese PLA General Hospital, Beijing, China; 2 Department of Clinical Radiology, Interdisciplinary Ultrasound-Center, University of Munich, Grosshadern Campus, Munich, Germany

**Keywords:** Gallbladder wall thickening, High frame rate contrast enhanced ultrasound, Gallbladder reporting and data system, Gallbladder carcinoma, Diagnosis

## Abstract

**Background:**

Ultrasound (US) has been widely used in screening and differential diagnosis of gallbladder wall thickening (GWT). However, the sensitivity and specificity for diagnosing wall-thickening type gallbladder cancer are limited, leading to delayed treatment or overtreatment. We aim to explore the value of high frame rate contrast enhanced ultrasound (H-CEUS) in distinguishing wall-thickening type gallbladder cancer (malignant) from GWT mimicking malignancy (benign).

**Methods:**

This retrospective study enrolled consecutive patients with non-acute GWT who underwent US and H-CEUS examination before cholecystectomy. Clinical information, US image and H-CEUS image characteristics between malignant and benign GWT were compared. The independent risk factors for malignant GWT on H-CEUS images were selected by multivariate logistic regression analysis. The diagnostic performance of H-CEUS in determining malignant GWT was compared with that of the gallbladder reporting and data system (GB-RADS) score.

**Results:**

Forty-six patients included 30 benign GWTs and 16 malignant GWTs. Only mural layering and interface with liver on US images were significantly different between malignant and benign GWT (*P* < 0.05). Differences in enhancement direction, vascular morphology, serous layer continuity, wash-out time and mural layering in the venous phase of GWT on H-CEUS images were significant between malignant and benign GWT (*P* < 0.05). The sensitivity, specificity and accuracy of H-CEUS based on enhancement direction, vascular morphology and wash-out time in the diagnosis of malignant GWT were 93.75%, 90.00%, and 91.30%, respectively. However, the sensitivity, specificity and accuracy of the GB-RADS score were only 68.75%, 73.33% and 71.74%, respectively. The area under ROC curve (AUC) of H-CEUS was significantly higher than that of the GB-RADS score (AUC = 0.965 vs. 0.756).

**Conclusions:**

H-CEUS can accurately detect enhancement direction, vascular morphology and wash-out time of GWT, with a higher diagnostic performance than the GB-RADS score in determining wall-thickening type gallbladder cancer. This study provides a novel imaging means with high accuracy for the diagnosis of wall-thickening type gallbladder cancer, thus may be better avoiding delayed treatment or overtreatment.

## Background

Gallbladder wall thickening (GWT) is a common clinical disease including primary and secondary disease, which refers to a thickness of gallbladder wall of more than 3.0 mm [1]. Primary GWT represents a wide spectrum of diseases, which are divided into benign and malignant diseases. The nature of GWT determines the management plan and type and extent of cholecystectomy. Misdiagnosis of GWT nature can lead to delayed treatment or extended cholecystectomy [1, 2]. Malignant GWT is usually a wall-thickening type gallbladder cancer, which accounts for approximately 20–30% of gallbladder cancers [3]. Due to nonspecific clinical symptoms in the early stage, less than 10% of patients can undergo radical cholecystectomy at first discovery, and the 5-year survival rate of advanced gallbladder cancer is only 5–15% [4]. Therefore, it is important to correctly distinguish wall-thickening type gallbladder cancer from benign GWT before selecting appropriate treatment.

Ultrasound (US) is the first-line imaging technique for detecting and diagnosing gallbladder disease due to its advantages of convenience, cost-effectiveness, radiation-free and real-time imaging [5, 6]. Some GWT diseases can be differentiated by US with high sensitivity, such as acute cholecystitis and gallbladder adenomyomatosis, because these diseases have specific US features [7–10]. However, there are still some difficulties in the differential diagnosis of non-acute GWT by US, especially in differentiating wall-thickening type gallbladder cancer and GWT mimicking malignancy (benign), which mainly includes chronic cholecystitis and xanthogranulomatous cholecystitis [11–13]. The gallbladder reporting and data system (GB-RADS) score has been constructed for risk stratification of GWT on US images [14]. However, the diagnostic performance of the GB-RADS score in distinguishing wall-thickening type gallbladder cancer from GWT mimicking malignancy needs to be further improved. There is also too much overlap of radiological imaging features (such as irregular gallbladder wall, discontinuous mucosa, enlarged lymph nodes and so on) to reliably differentiate wall-thickening type gallbladder cancer and GWT mimicking malignancy [15, 16].

Contrast enhanced ultrasound (CEUS) can accurately detect microcirculation characteristics and has been used in the diagnosis of gallbladder diseases, such as sludge, gallbladder polyps and gallbladder cancer [5, 17, 18]. However, both wall-thickening type gallbladder cancer and GWT mimicking malignancy could present similar enhancement features, including inhomogeneous hyperenhancement intensity and destroyed serous layer on CEUS images, which poses great challenges for differential diagnosis [19, 20]. Therefore, a new imaging technique is required to distinguish wall-thickening type gallbladder cancer from GWT mimicking malignancy. Compared with CEUS, high frame rate CEUS (H-CEUS) has higher temporal resolution and can more accurately reflect blood perfusion features of focal liver lesions and gallbladder polyps, which improves the accuracy of disease diagnosis [21, 22]. We speculated that H-CEUS could reflect a more detailed dynamic enhancement process of GWT and improve the differential diagnostic performance of non-acute GWT. To the best of our knowledge, there is no research report on H-CEUS distinguishing wall-thickening type gallbladder cancer from GWT mimicking malignancy at present.

In this study, we compared H-CEUS image features between wall-thickening type gallbladder cancer and GWT mimicking malignancy to determine independent factors related to malignant GWT, and explore the diagnostic performance of H-CEUS compared to the GB-RADS score to provide more valuable diagnostic information for patients with non-acute GWT to select appropriate treatment.

## Methods

### Participants

**Fig. 1:**
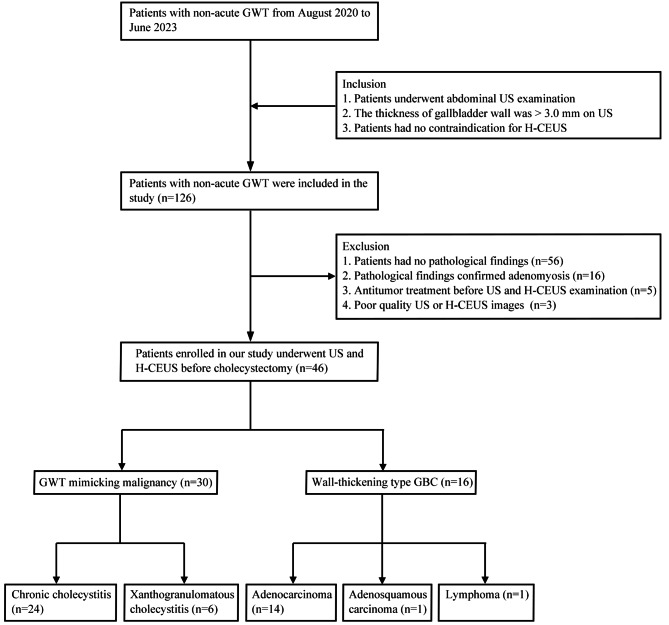
Study flowchart. GWT, gallbladder wall thickening; US, ultrasound; H-CEUS, high frame rate contrast enhanced ultrasound; GBC, gallbladder carcinoma

This study involving human participants was approved by the Ethics Committees of Chinese PLA General Hospital. From August 2020 to June 2023, 126 consecutive patients with non-acute GWT were retrospectively reviewed. The inclusion criteria were as follows: (1) patients underwent abdominal US examination; (2) the thickness of gallbladder wall was > 3.0 mm on US; and (3) patients had no contraindications for H-CEUS. The exclusion criteria were as follows: (1) patients had no pathological findings (*n* = 56); (2) pathological findings confirmed adenomyomatosis (*n* = 16); (3) antitumor treatment before US and H-CEUS examination (*n* = 5); and (4) poor quality US or H-CEUS images (*n* = 3). A total of 46 patients were finally enrolled in our study (Fig. 1).

### US equipment and scanning protocol

Resona R9 (Mindray, Shenzhen, China) was used to perform US and H-CEUS scanning. The convex transducer SC5-1U (frequency 2–5 MHz) was used. All US examinations were performed by a physician with more than 15 years of experience in abdominal CEUS. After fasting for more than 8 h, all patients underwent continuous US and H-CEUS examinations. During the US scanning, gallbladder and adjacent liver tissues were observed. The H-CEUS software was ultrawide nonlinear, and the frame rate was more than 50 Hz. A low mechanical index ranging from 0.06 to 0.09 was used for real-time H-CEUS. SonoVue (Bracco, Milan, Italy) was used as the US contrast agent. After intravenous injection of contrast agent (0.02 mL/kg), 10.0 mL of saline flushed the intravenous catheter. All US images were saved in DICOM format for playback and analysis. No adverse side effects upon administration of SonoVue were registered.

### Clinical information and US image analysis

The patient’s age, sex and preoperative serum examination including routine blood (leukocyte, neutrophil and C-reactive protein level) and tumor marker (CEA, AFP, CA125, CA19-9, CA15-3 and CA72-4) examination were obtained from medical records. Two physicians with more than 10 years of experience in abdominal CEUS independently interpreted the characteristics of US and H-CEUS images. Both physicians were blind to the clinical information and pathology findings. If the two physicians had different conclusions, a third physician with more than 15 years of experience in abdominal CEUS who performed all US examinations made the final decision after discussion. Interobserver agreements between the two physicians were evaluated with intraclass correlation coefficients or kappa coefficients.

The characteristics of US images include the following [14]: (1) gallstone (present or absent); (2) the thickness of GWT; (3) extent of GWT (focal or circumferential); (4) symmetry of GWT (symmetric or asymmetric); (5) mural layering (present or absent); (6) intramural changes (including intramural cysts and echogenic foci, present or absent); (7) interface with liver (distinct or indistinct); (8) echogenicity (hyper, iso, and hypo, compared to normal liver parenchyma more than 5 cm away from gallbladder); (9) echogenicity homogeneity (homogeneous or inhomogeneous); and (10) vascularity detected by color Doppler flow imaging (present or absent). GB-RADS score was evaluated as following: GB-RADS 2, Symmetric circumferential thickening with or without intramural changes or focal thickening with intramural changes, layered appearance, and distinct interface with liver; GB-RADS 3, Circumferential thickening without layered appearance, focal thickening without intramural features (cysts or echogenic foci) or layered appearance, and distinct interface with liver; GB-RADS 4, Circumferential or focal thickening without layered appearance and with loss of interface with liver; GB-RADS 5, Same as GB-RADS 4 with definite extramural invasion, such as biliary or vascular involvement or liver mass [14].

### H-CEUS image characteristics analysis

Gallbladder CEUS is divided into two phases: the arterial phase starts from around 10–20 s until 30 s after contrast agent injection, and the venous phase starts from 30 to 120 s after contrast agent injection [17]. The characteristics of H-CEUS image were as follows: (1) wash-in time of GWT; (2) enhancement direction of GWT (inner to outer or outer to inner); (3) enhancement intensity (hyper or non-hyper, compared to normal liver parenchyma in the arterial phase); (4) vascular morphology of GWT (regular or irregular); (5) enhancement homogeneity (homogeneous or inhomogeneous); (6) serous layer continuity (intact or destroyed); (7) wash-out time of GWT; (8) mural layering in the venous phase (present or absent); (9) wash-in time of adjacent liver that surrounding gallbladder (earlier or non-earlier, compared to normal liver parenchyma more than 5 cm away from gallbladder); (10) enhancement intensity of adjacent liver in the arterial phase (hyper or non-hyper); (11) wash-out time of adjacent liver (earlier or non-earlier); and (12) enhancement intensity of adjacent liver in the venous phase (hypo or non-hypo).

### Statistical analysis

Statistical analysis was performed with SPSS 26.0 (IBM Corporation, Armonk, NY, USA). All quantitative parameters are expressed as the mean ± standard deviation. The *t-*test was used only for normally distributed quantitative parameters, and the Mann-Whitney *U* test was used for nonnormally distributed quantitative parameters. Comparisons of categorical data were performed with the chi-square test. Receiver operating characteristic (ROC) curve analysis was used to evaluate the cutoff value of continuous variables and the sensitivity, specificity and accuracy of the GB-RADS score and H-CEUS in the differential diagnosis of non-acute GWT. The potential risk variables of malignant GWT were screened by univariate logistic analysis. Variables with a *P* value < 0.01 were used to further identify the independent factors for malignant GWT by multivariate logistic regression. A *P* value less than 0.05 indicated statistical significance.

## Results

### Pathology findings and clinical information

In this study, 30 cases were benign GWT and 16 cases were malignant GWT. Benign GWT included 24 cases of chronic cholecystitis and 6 cases of xanthogranulomatous cholecystitis. Among the malignant GWT, there were 14 cases of gallbladder adenocarcinoma, 1 case of adenosquamous carcinoma and 1 case of lymphoma. The average age of all patients was 54.37 ± 12.58 years, ranging from 25 years to 74 years. Patients with malignant GWT were significantly older than those with benign GWT (*P* = 0.002). There were 23 males and 23 females in the study. Gender was not different between malignant GWT and benign GWT (*P* = 0.216). Neutrophil in malignant GWT was significantly higher than that in benign GWT (*P* = 0.007), while leukocyte and C-reactive protein levels were not different between malignant GWT and benign GWT (*P* = 0.094 and 0.196, respectively). Tumor marker examination (CEA, AFP, CA125, CA19-9, CA15-3 and CA72-4) between malignant GWT and benign GWT were not different (*P* = 0.344, 0.204, 0.556, 0.661, 0.393 and 0.612, respectively) (Table 1).

**Table 1:** Serum examination of non-acute gallbladder wall thickening

Characteristics	Normal value	Benign (30)	Malignant (16)	t /z	*P*
Leukocyte (10^9^/L)	3.5–10	6.30 ± 2.48	6.92 ± 1.82	-1.672	0.094
Neutrophil (%)	50–70	55.80 ± 11.79	66.13 ± 12.10	-2.803	0.007
C-reactive protein (mg/dL)	0-0.8	1.47 ± 2.40	1.77 ± 1.82	-1.293	0.196
CEA (ug/L)	0–5.0	2.03 ± 1.09	3.05 ± 3.08	-0.946	0.344
AFP (ug/L)	0–20	3.17 ± 1.01	3.62 ± 1.32	-1.289	0.204
CA125 (u/mL)	0.1–35	14.07 ± 7.03	39.41 ± 83.29	-0.588	0.556
CA19-9 (u/mL)	0.1–37	60.21 ± 167.29	156.72 ± 463.10	-0.438	0.661
CA15-3 (u/mL)	0.1–30	12.59 ± 4.71	26.16 ± 36.10	-0.854	0.393
CA72-4 (u/mL)	0.1–10	5.29 ± 5.77	3.30 ± 1.67	-0.508	0.612

### US image characteristics

Gallstones could be seen in 30 cases of non-acute GWT, and there was no difference between malignant GWT and benign GWT (*P* > 0.05). The thickness of GWT ranged from 3.6 to 27.4 mm, and gallbladder wall thickness was not different between malignant GWT and benign GWT (*P* > 0.05). According to the GB-RADS score, the US image features were compared in non-acute GWT (Table 2). Only mural layering and interface with liver were significantly different between malignant GWT and benign GWT (*P* < 0.05). The absence of mural layering and indistinct interface with liver were more common in malignant GWT than in benign GWT. The extent of involvement, symmetry and intramural changes in GWT between malignant GWT and benign GWT were not different (*P* > 0.05). In addition, there was no difference in echogenicity, echogenicity homogeneity or vascularity of GWT between malignant GWT and benign GWT (*P* > 0.05). Interobserver agreements of US image characteristics ranged from 0.713 to 0.856.

**Table 2:** US image characteristics of non-acute gallbladder wall thickening

Characteristics	Benign (30)	Malignant (16)	z/χ^2^	*P*
Gallstone			0.080	0.777
Present	20	10		
Absent	10	6		
Gallbladder wall thickness (mm)	8.46 ± 5.01	9.27 ± 3.51	-1.280	0.200
Extent of involvement			2.807	0.094
Focal	11	10		
Circumferential	19	6		
Symmetry of wall thickening			2.690	0.101
Symmetric	15	4		
Asymmetric	15	12		
Mural layering			5.148	0.023
Present	16	3		
Absent	14	13		
Intramural changes			3.460	0.130
Present	9	1		
Absent	21	15		
Interface with liver			5.625	0.018
Distinct	22	6		
Indistinct	8	10		
Echogenicity			0.484	0.925
Hyper	9	6		
Iso	9	5		
Hypo	12	5		
Echogenicity homogeneity			0.163	0.686
Homogeneous	15	7		
Inhomogeneous	15	9		
Vascularity			3.490	0.062
Present	16	13		
Absent	14	3		

US, ultrasound

### H-CEUS image characteristics

**Fig. 2:**
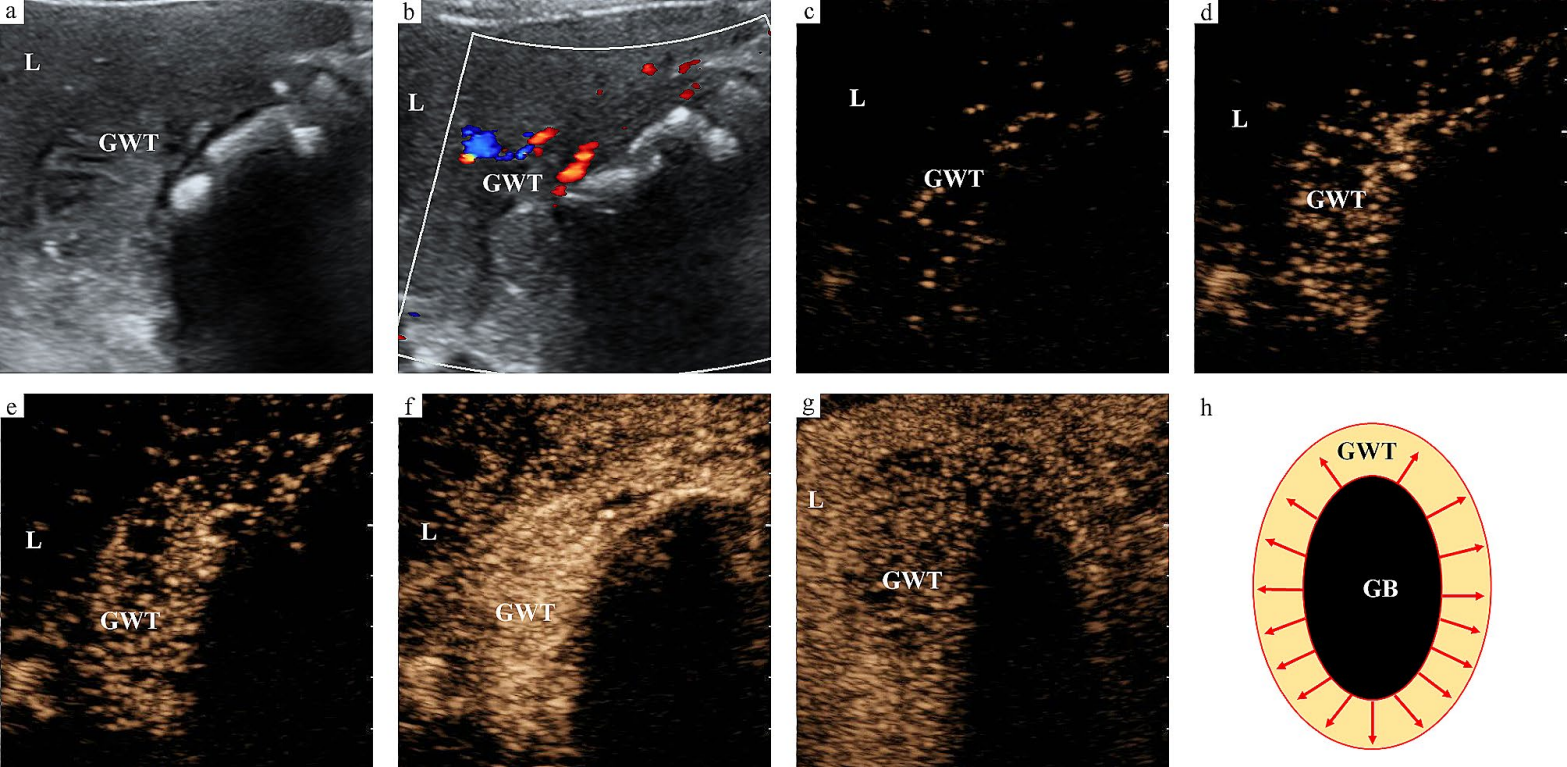
US images of xanthogranulomatous cholecystitis. (**a**) Thickening of gallbladder wall on B-mode US image (GB-RADS 4). (**b**) Vascularity in GWT on color Doppler flow imaging. (**c-f**) Enhancement direction of GWT is from inner to outer, with regular vascular morphology. (**g**) Wash-out time is 70 s. (**h**) Schematic diagram of the enhancement pattern of GWT mimicking malignancy on H-CEUS images. US, ultrasound; H-CEUS, high frame rate contrast enhanced ultrasound; GWT, gallbladder wall thickening; L, liver; GB, gallbladder

**Fig. 3:**
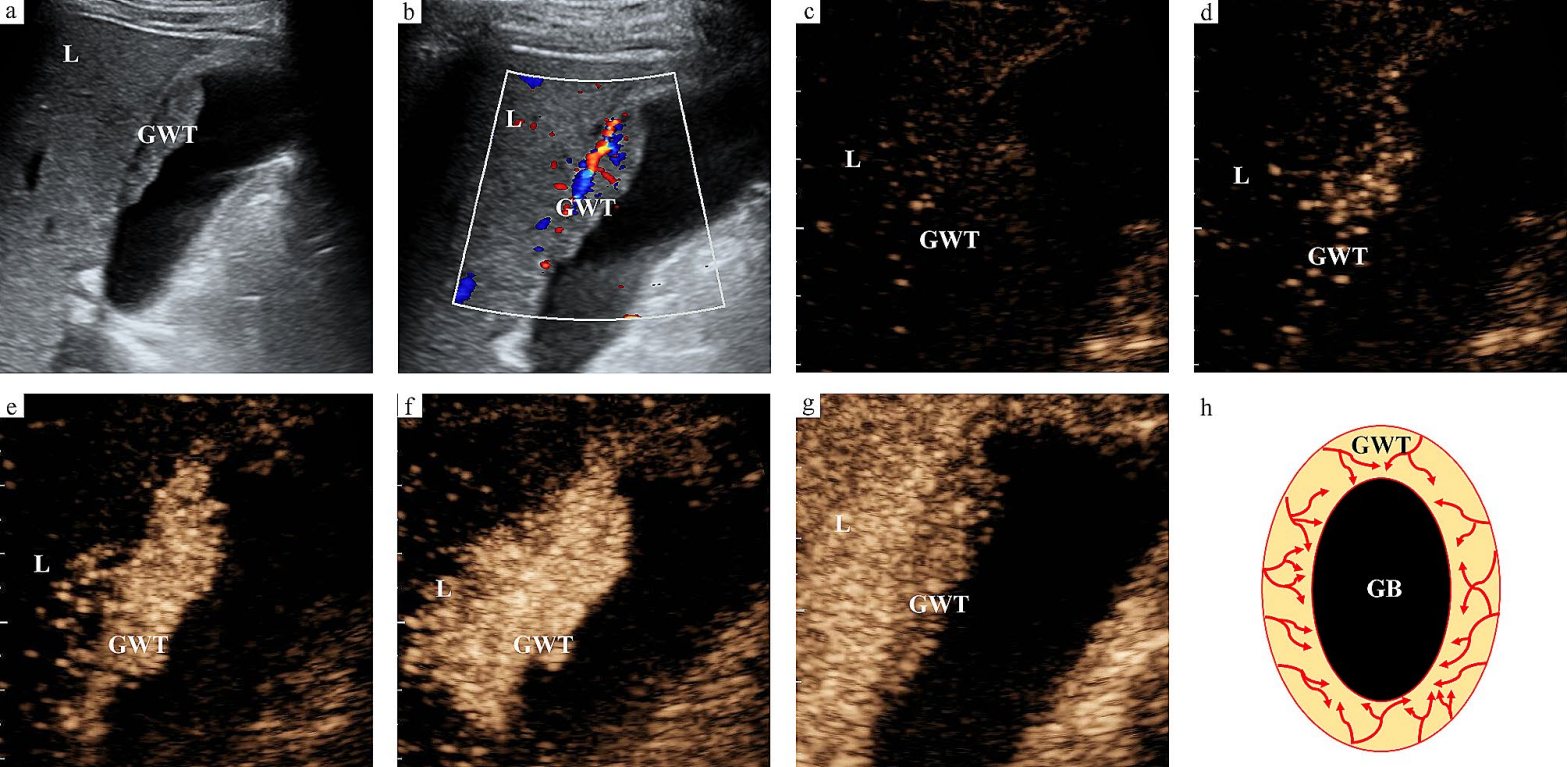
US images of gallbladder adenocarcinoma. (**a**) Thickening of gallbladder wall on B-mode US image (GB-RADS 4). (**b**) Vascularity in GWT on color Doppler flow imaging. (**c-f**) Enhancement direction of GWT is from outer to inner, with irregular vascular morphology. (**g**) Wash-out time is 37 s. (**h**) Schematic diagram of the enhancement pattern of wall-thickening type gallbladder cancer on H-CEUS images. US, ultrasound; H-CEUS, high frame rate contrast enhanced ultrasound; GWT, gallbladder wall thickening; L, liver; GB, gallbladder

The enhancement direction of all malignant GWTs was from outer to inner. However, the enhancement direction of benign GWT included not only from inner to outer, but also from outer to inner. The enhancement direction of malignant GWT was significantly different from that of benign GWT (*P* < 0.05) (Figs. 2 and 3). The proportion of irregular vascular morphology in malignant GWT was significantly higher than that in benign GWT, while the proportion of regular vascular morphology in benign GWT was significantly higher than that in malignant GWT (*P* < 0.05) (Figs. 2 and 3). A destroyed serous layer could be seen in most malignant GWTs, while the majority of benign GWTs had an intact serous layer, and the difference between malignant GWT and benign GWT was significant (*P* < 0.05). The wash-out time in malignant GWT was significantly shorter than that in benign GWT (*P* < 0.05) (Figs. 2 and 3). Based on the ROC analysis results, the optimal cutoff value of wash-out time for predicting the nature of non-acute GWT was 42 s, and the wash-out time of malignant GWT was usually not more than 42 s. Malignant GWT usually had no mural layering in the venous phase, while most benign GWTs had mural layering in the venous phase, and the difference between malignant GWT and benign GWT was significant (*P* < 0.05). The wash-in time, enhancement intensity and enhancement homogeneity of GWT were not different between malignant GWT and benign GWT (*P* > 0.05) (Table 3). The enhancement characteristics of adjacent liver parenchyma were also evaluated. The wash-in time of adjacent liver parenchyma was earlier than that of normal liver parenchyma, which could be seen in both malignant GWT and benign GWT, and the proportion of malignant GWT was higher. However, the wash-in time and enhancement intensity in the arterial phase of adjacent liver parenchyma were not different between malignant GWT and benign GWT (*P* > 0.05). The wash-out time and enhancement intensity in the venous phase of adjacent liver parenchyma were significantly different between malignant GWT and benign GWT (*P* < 0.05). The early wash-out time and hypo enhancement intensity in the venous phase of adjacent liver parenchyma were more common in malignant GWT than in benign GWT. Interobserver agreements of H-CEUS image characteristics ranged from 0.731 to 0.863.

**Table 3:** H-CEUS image characteristics of non-acute gallbladder wall thickening

Characteristics	Benign (30)	Malignant (16)	t /z/χ^2^	*P*
Wash-in time (s)	11.80 ± 2.47	12.81 ± 1.83	-1.821	0.069
Enhancement direction			14.382	< 0.001
Inner to outer	17	0		
Outer to inner	13	16		
Enhancement intensity			1.529	0.394
Hyper	24	15		
Non-hyper	6	1		
Vascular morphology			15.476	< 0.001
Regular	22	2		
Irregular	8	14		
Enhancement homogeneity			1.217	0.270
Homogeneous	20	8		
Inhomogeneous	10	8		
Serous layer			10.984	0.001
Intact	21	3		
Destroyed	9	13		
Wash-out time (s)	57.37 ± 12.98	38.50 ± 9.39	5.132	< 0.001
> 42	26	3	20.659	< 0.001
≤ 42	4	13		
Mural layering in the venous phase			8.396	0.004
Present	17	2		
Absent	13	14		
Adjacent liver wash-in time			2.054	0.189
Earlier	7	7		
Non-earlier	23	9		
Adjacent liver enhancement intensity in the arterial phase			2.054	0.189
Hyper	7	8		
Non-hyper	23	8		
Adjacent liver wash-out time			7.170	0.015
Earlier	1	5		
Non-earlier	29	11		
Adjacent liver enhancement intensity in the venous phase			7.170	0.015
Hypo	1	5		
Non-hypo	29	11		

H-CEUS, high frame rate contrast enhanced ultrasound

### Diagnostic performance of GB-RADS and H-CEUS

**Fig. 4:**
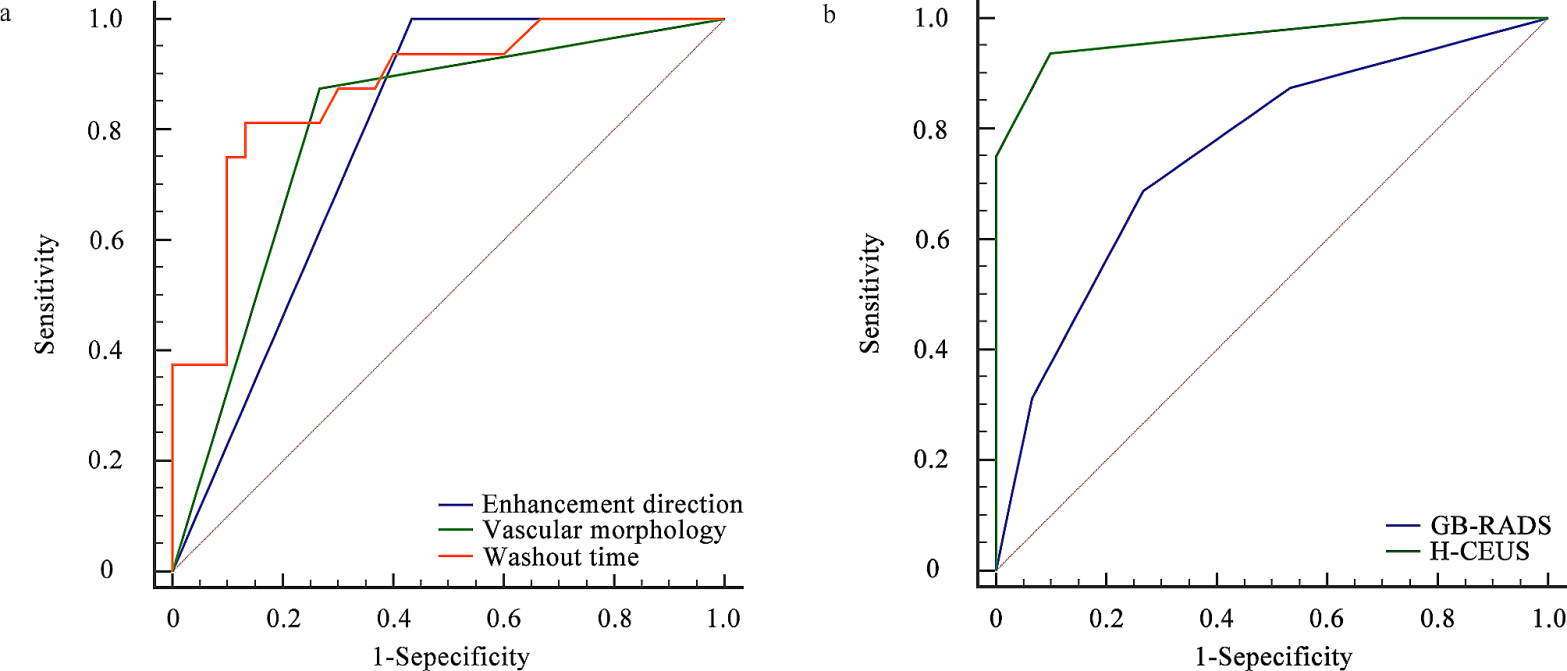
ROC curve analysis of diagnostic performance. (**a**) Diagnostic performance of H-CEUS parameters (enhancement direction, vascular morphology and wash-out time) in distinguishing wall-thickening type gallbladder cancer. (**b**) Diagnostic performance of the GB-RADS score and H-CEUS in distinguishing wall-thickening type gallbladder cancer. H-CEUS, high frame rate contrast enhanced ultrasound; GB-RADS, gallbladder reporting and data system

The optimal cutoff value of the GB-RADS score for predicting malignant GWT was 4 (Table 4). Vascular morphology and wash-out time of GWT were significant independent predictors of malignant GWT (Table 5). Considering that the sensitivity of enhancement direction to diagnose malignant GWT was 100%, we distinguished malignant GWT and benign GWT based on the enhancement direction, vascular morphology and wash-out time on H-CEUS images (Table 6; Fig. 4a). Among the three parameters, two or more parameters were met, and H-CEUS was diagnosed as malignant GWT. The sensitivity, specificity, accuracy and AUC of the GB-RADS score and H-CEUS in distinguishing non-acute GWT are shown in Table 6. The diagnostic performance of H-CEUS was significantly better than that of the GB-RADS score in distinguishing malignant GWT from benign GWT (*P* < 0.05) (Fig. 4b).

**Table 4:** GB-RADS score of non-acute gallbladder wall thickening

GB-RADS score	2	3	4	5
Benign (30)	14	8	6	2
Malignant (16)	2	3	6	5

**Table 5:** Independent risk factors for wall-thickening type gallbladder cancer

Parameters	Coefficient	Standard error	Odds ratio	95% confidence interval	*P*
Vascular morphology	3.145	1.114	23.223	2.616 -206.145	0.005
Wash-out time	-0.151	0.052	0.860	0.777–0.952	0.004

**Table 6:** Diagnostic performance of US parameters to determine wall-thickening type gallbladder cancer

Ultrasound parameter	Sensitivity (%)	Specificity (%)	Accuracy (%)	AUC
Enhancement direction	100.00	56.67	71.74	0.783
Vascular morphology	87.50	73.33	78.26	0.804
Wash-out time	81.25	86.67	84.78	0.873
GB-RADS	68.75	73.33	71.74	0.756
H-CEUS	93.75	90.00	91.30	0.965

US, ultrasound; GB-RADS, gallbladder reporting and data system; H-CEUS, high frame rate contrast enhanced ultrasound

## Discussion

Distinguishing wall-thickening type gallbladder cancer from GWT mimicking malignancy still poses a great challenge for physicians. The sensitivity of preoperative imaging diagnosis for wall-thickening type gallbladder cancer is less than 80% [12, 16]. Therefore, discovering new imaging features through new imaging techniques is the key to improving the diagnostic performance of wall-thickening type gallbladder cancer. H-CEUS is a novel US imaging technique and has the advantage of higher temporal resolution, which could more accurately demonstrate information on the dynamic perfusion process of GWT. In this study, H-CEUS was firstly used to distinguish malignant GWT from benign GWT and had better diagnostic performance than the GB-RADS score. On H-CEUS images, we firstly found that the enhancement direction and vascular morphology of GWT were valuable in distinguishing malignant GWT from benign GWT, which is different from previous studies.

Compared to CT or MRI, CEUS is a real-time imaging technique and perfusion process can be displayed. Therefore, the enhancement direction was firstly evaluated in this study, which was few studies reported before. The enhancement direction in all malignant GWTs was from outer to inner, while in most benign GWTs the enhancement direction was from inner to outer on H-CEUS images. Therefore, enhancement direction was an important parameter for predicting malignant GWT, which was related with the pathological characteristics. The nourishing vessels of normal gallbladder wall from gallbladder neck run along serous layer of gallbladder wall and are distributed towards mucosal layer. Gallbladder cancer originates from mucosal layer and grows invasively to serous layer. The nourishing vessels of malignant GWT were distributed from serous layer to mucosal layer [23], so the enhancement direction was from outer to inner on H-CEUS images, that is, from serous layer to mucosal layer. The mucosal layer of GWT mimicking malignancy becomes thinner or even disappears due to inflammation, and serous layer becomes thicker due to the proliferation of fibrous tissue. Only the thickened serous layer was shown on H-CEUS images, so the enhancement direction from inner to outer could be seen in benign GWT. When the internal structure of GWT changed little, the enhancement direction was still from outer to inner in benign GWT on H-CEUS images. Therefore, the two types of enhancement directions on H-CEUS images could both be seen in benign GWT. The perfusion process of CEUS in GWT is fast, and accurate evaluation of the enhancement direction requires a high frame rate. Compared to conventional CEUS, H-CEUS has a higher frame rate and can offer more enhancement information, which could be used to reflect the enhancement direction difference of GWT. The enhancement direction from inner to outer of gallbladder wall could only be seen in benign GWT, which was beneficial for the differential diagnosis of non-acute GWT.

H-CEUS improves the temporal resolution of CEUS and can more accurately reflect the vascular morphology of tissues and lesions [21, 22]. On H-CEUS images, the vascular morphology of malignant GWT was mostly irregular, while the majority of benign GWTs showed regular vascular morphology. The abnormal proliferation and distortion of blood vessels form a disordered distribution of blood vessels in malignant GWT, which leads to irregular vascular morphology on H-CEUS images [13]. Benign GWT presented as artery dilation and vein filling in GWT, but the vascular network was arranged relatively regularly, so regular (comb-like) vascular morphology could be seen on H-CEUS images [13]. Therefore, the vascular morphology of GWT on H-CEUS images could be used as an important feature to distinguish malignant GWT and benign GWT.

Early wash-out has been widely used to distinguish gallbladder cancer from benign gallbladder diseases. A large number of arteriovenous fistulas were present in malignant GWT, so it presented as early wash-out. The washout time in malignant GWT was shorter than that in benign GWT in our study. The wash-out time of most malignant GWTs was not more than 42 s, while the majority of benign GWTs was more than 42 s, which was similar to previous studies [13, 18]. Our finding further confirmed that early wash-out on H-CEUS images was associated with malignant GWT.

The GB-RADS score based on US image features of gallbladder wall is proposed to improve consistency in the assessment of risk of malignancy in non-acute GWT [14]. Mural layering, intramural changes and interface with liver are the key parameters of the GB-RADS score that are used to evaluate malignant GWT. In this study, mural layering and interface with liver were significantly different between malignant GWT and benign GWT. However, the difference in intramural changes between malignant GWT and benign GWT was not significant, which might be because our study did not include gallbladder adenomyomatosis. Gallbladder adenomyomatosis shows intramural changes (intramural cysts and echogenic foci) in GWT on US images, so it is relatively easier to distinguish gallbladder adenomyomatosis from other types of GWT [24]. Our study aimed to differentiate wall-thickening type gallbladder cancer and GWT mimicking malignancy, which was a great challenge to the GB-RADS score. H-CEUS could accurately reflect the microcirculation perfusion process of GWT, thus improving the diagnostic performance of wall-thickening type gallbladder cancer compared to the GB-RADS score.

Age is a risk factor for gallbladder cancer, and the risk of gallbladder cancer increases with age. Our study also found that patients with malignant GWT were older than those with benign GWT, which was consistent with previous reports [19]. CA19-9 has been used in the diagnosis and prognosis evaluation of patients with bile duct carcinomas [4]. The level of CA19-9 in malignant GWT and benign GWT both increased, and the difference was not significant in our study. This might be because our study included six cases of xanthogranulomatous cholecystitis, which also increased the level of CA19-9 [25]. Gallbladder cancer is highly invasive and rapidly grows deep into gallbladder wall, leading to GWT. Fibrous tissue proliferation of chronic cholecystitis and invasive growth of xanthogranulomatous cholecystitis can both lead to GWT [25]. Therefore, both malignant diseases and benign diseases can cause thickening of gallbladder wall. Our study also found that the thickness of gallbladder wall was not different between malignant GWT and benign GWT, which could not be used to distinguish wall-thickening type gallbladder cancer.

This study has the following limitations: (1) This was a single-center retrospective study. A prospective multi-center study with a larger sample can further clarify the value of H-CEUS in the differential diagnosis of wall-thickening type gallbladder cancer and GWT mimicking malignancy. Deep learning based on H-CEUS may further improve the diagnostic performance of wall-thickening type gallbladder cancer [26]. (2) The overall sample size and patient numbers in subgroups were small, but comparable to patient numbers in similar studies. We have adopted a reasonable research design and appropriate statistical methods, so the results and conclusions are reliable. Differentiating wall-thickening type gallbladder cancer from GWT mimicking malignancy is critical as it may help avert poor prognosis.

## Conclusions

Enhancement direction, vascular morphology and wash-out time of GWT were valuable features for distinguishing malignant GWT from benign GWT by H-CEUS. Compared with the GB-RADS score, the diagnostic performance was better on H-CEUS in distinguishing wall-thickening type gallbladder cancer from GWT mimicking malignancy. Therefore, H-CEUS would help obtain more valuable diagnostic information to choose appropriate treatment for patients with non-acute GWT.

## Data Availability

The data that support the findings of this study are available from the corresponding author upon reasonable request.

## Abbreviations

AUC: Area under the receiver operating characteristic curve
CEUS: Contrast enhanced ultrasound
GB-RADS: Gallbladder reporting and data system
GWT: Gallbladder wall thickening
H-CEUS: High frame rate contrast enhanced ultrasound
ROC: Receiver operating characteristic
US: Ultrasound
